# Association of Oxidative-Stress-Related Gene Polymorphisms with Pain-Related Temporomandibular Disorders and Oral Behavioural Habits

**DOI:** 10.3390/antiox12061195

**Published:** 2023-05-31

**Authors:** Ema Vrbanović, Marko Zlendić, Koraljka Gall Trošelj, Marko Tomljanović, Kristina Vuković Đerfi, Iva Z. Alajbeg

**Affiliations:** 1 Department of Removable Prosthodontics, School of Dental Medicine, University of Zagreb, Gundulićeva 5, 10000 Zagreb, Croatia; evrbanovic@sfzg.hr (E.V.); mzlendic@sfzg.hr (M.Z.); 2 Laboratory for Epigenomics, Division of Molecular Medicine, Ruđer Bošković Institute, 10000 Zagreb, Croatia; troselj@irb.hr (K.G.T.); marko.tomljanovic@irb.hr (M.T.); 3 Laboratory for Personalized Medicine, Division of Molecular Medicine, Ruđer Bošković Institute, 10000 Zagreb, Croatia; kvukovic@irb.hr; 4 Department of Dentistry, Clinical Hospital Center Zagreb, 10000 Zagreb, Croatia

**Keywords:** chronic orofacial pain, oxidative-stress-related genes, temporomandibular disorders, oral behavioural habits, single-nucleotide polymorphism

## Abstract

The frequency of selected polymorphisms, one in each gene coding for proteins with antioxidative properties (*CAT*(rs1001179), *SOD2*(rs4880), *GPX1*(rs1050450), and *NQO1*(rs689452)), was compared between patients suffering from pain-related temporomandibular disorders (TMDp; *n* = 85) and control subjects (CTR; *n* = 85). The same was evaluated when participants were divided with respect to oral behavioural habits frequency into high-frequency parafunction (HFP; *n* = 98) and low-frequency parafunction (LFP; *n* = 72) groups. Another aim was to investigate whether polymorphisms in these genes can be associated with participants’ psychological and psychosomatic characteristics. Polymorphisms were genotyped using the genomic DNA extracted from buccal mucosa swabs and real-time TaqMan genotyping assays. No differences in genotype distribution between TMDp patients and control subjects were found. Still, TMDp patients who were homozygous for minor allele A, related to the *GPX1* polymorphism rs1050450, reported significantly more waking-state oral behaviours than GA + GG genotype carriers (score: 30 vs. 23, *p* = 0.019). The frequency of genotype AA for rs1050450 polymorphism was higher in HFP than in LFP participants (14.3% vs. 4.2%, *p*  = 0.030). The most important predictors of waking-state oral behaviours were depression, anxiety, AA genotype (rs1050450), and female sex. The explored gene polymorphisms were not found to be significant risk factors for either TMDp or sleep-related oral behaviours. The association of waking-state oral behaviours with selected gene polymorphisms additionally supports previous assumptions that daytime bruxism is more closely linked to various stress manifestations, which might also be reflected through the variability related to the cellular antioxidative activity.

## 1. Introduction

Temporomandibular disorders (TMD) are the most common cause of chronic pain of non-dental origin in the orofacial region and the second most common musculoskeletal disorder that causes pain and disability [1]. They are defined by a multifactorial aetiology explained through the biopsychosocial model that defines pain and disability as a multifaceted, dynamic integration of physiological, psychological, and social risk factors that interact and are influenced by the environment and genetics. TMD symptoms affect a significant number of people worldwide (incidence: 3.9%; prevalence: 3.7%) [2,3,4]. As a chronic pain condition, the symptoms sometimes might overlap with other chronic disorders such as headache, fibromyalgia, and neurological conditions, probably through the phenomenon of central sensitisation, mainly allodynia and hyperalgesia [5].

TMD symptoms can be categorised into masticatory muscle-related symptoms and temporomandibular joint (TMJ)-related symptoms. Clinically, there is pain in these areas, limitations in jaw movements, and occurrence of joint sounds. Diagnostic Criteria for Temporomandibular Disorders (DC/TMD), a validated clinical practice and research protocol, consists of two subprotocols [6]. 

Axis I subprotocol includes a reliable screener for detecting any pain-related TMD (TMDp), as well as valid diagnostic criteria for distinguishing pain-related TMD from TMJ disorders (disc displacements, degenerative joint diseases, and subluxation). Pain-related TMD includes masticatory muscle myalgia, arthralgia (which refers to TMJ pain), and headache attributed to TMD [6]. 

Axis II, on the other hand, consists of screening and comprehensive self-report instruments assessing pain intensity, jaw functional limitations, psychological distress, pain-related disability, and oral behavioural habits. Painful symptoms in TMD frequently coexist and might be aggravated by parafunctional behaviours and activities of the mouth, beyond its original functions of chewing, talking, and swallowing [7]. They include bruxism, repetitive muscle activity that is accompanied by clenching or grinding the teeth and/or pushing the lower jaw while awake or during sleep [8]. Psychological variables (such as anxiety and depression) and patients’ susceptibility to stress are thought to be relevant in the aetiology of both oral behaviours (OBs) and TMD [9]. Therefore, the overlapping background of these two disorders and the undoubted influence of stress depending on patients’ psychological profile is the reason why the connection between OBs and TMD is frequently studied [10].

Oxidative stress, defined as an imbalance between the formation of reactive oxygen species (ROS) and antioxidant defence, has been implicated in the development and progression of a variety of pathological conditions, including chronic pain disorders such as TMD [11]. Prolonged exposure to exogenous stress and psychological stressors leads to the increased production of markers of oxidative DNA damage [12]. For example, hypoxia reduces blood antioxidants level while increasing the markers of oxidative stress (for example, malondialdehyde level, as shown in rat tissues), leading to a higher chance of oxidative damage and subsequent pathological alterations in the body [13]. 

The successful removal of ROS is regulated by the enzymatic antioxidant system, involving proteins with enzymatic antioxidative properties, which are coded by corresponding genes. Particular single-nucleotide polymorphisms (SNPs) in those genes can alter the activity of key antioxidant enzymes, thus promoting imbalances in the cellular oxido-redox status. Specific genetic variability may predispose individuals to the development and severity of certain diseases and influence susceptibility to environmental factors [14]. 

Although an impaired oxidative protective mechanism has been suggested as an aetiological risk factor for pain disorders such as fibromyalgia, headaches, and TMD, there has been limited research on SNPs of genes coding for proteins with antioxidative properties involved in these disorders [15,16]. So far, studies have shown that individuals with TMD tend to have higher salivary and serum levels of oxidative stress markers, such as malondialdehyde, and lower levels of specific antioxidants with a consequential decrease in antioxidant capacity compared with healthy controls [17,18]. By contrast, some studies have proposed a compensatory increase in the antioxidant defence, with higher salivary total antioxidant capacity in combination with higher salivary levels of oxidants in TMD patients compared with controls [19,20]. Because TMD is linked to anxiety and depression, hypervigilance, and a tendency to somatisation, it is important to note that oxidative damage might be involved in nervous system dysfunction. One research found that the expression of the genes involved in antioxidative metabolism (glutathione reductase 1 and glyoxalase 1) correlate with anxiety-related traits, with antioxidant enzyme activity being highest in the most anxious mice and lowest in the least anxious animals [21].

The cause-and-effect link between chronic pain, OBs, psychological stress, and oxidative stress is currently debated, and further research is needed for a better understanding of the precise mechanisms through which these conditions influence response to oxidative stress and/or vice versa. The presence of SNPs in the genes encoding enzymes with antioxidative properties has been associated with various conditions. They may also be associated with TMD and OBs [22,23,24]. Furthermore, since certain patients are more influenced by aggravating factors such as psychological stress and OBs, it is important to investigate the gene–behaviour associations in TMD patients.

This study aimed to investigate the distribution of SNPs in the genes coding for antioxidant enzymes, namely catalase (*CAT*), superoxide dismutase 2 (*SOD2*), glutathione peroxidase 1 (*GPX1*), and NAD(P)H quinone dehydrogenase 1 (*NQO1*), in TMDp patients and healthy controls. The distribution of SNPs was also evaluated with respect to oral behavioural habits. Another aim was to investigate whether SNPs in these genes can be associated with participants’ psychological and psychosomatic characteristics. 

The null hypothesis stated that the investigated SNPs of interest would not be associated with the presence of pain-related TMD or the frequency of harmful oral behaviours.

## 2. Materials and Methods

This case–control research was carried out at the University of Zagreb, School of Dental Medicine, in accordance with the Declaration of Helsinki’s ethical norms. The study protocol was approved by the Ethics Committee at the School of Dental Medicine, University of Zagreb (05-PA-30-VIII-6/2019). The trial was filed as NCT046 on ClinicalTrials.gov on 4 January 2021. The study protocol was written in conformity with the STROBE Statement: Guidelines for Reporting Observational Studies in Epidemiology [25]. Furthermore, the Strengthening the Reporting of Genetic Association Studies (STREGA) standards were adhered to (Supplementary Materials) [26]. Before participating in the study, each subject had to sign an informed consent form.

From January 2020 to September 2022, 85 subjects (76 females, 9 males) were selected from the group of patients sent to the Department of Dentistry at the Clinical Hospital Centre Zagreb due to chronic orofacial pain. Participants had to be over the age of 18 and had a confirmed diagnosis of TMDp (myalgia and/or arthralgia), according to DC/TMD. The average pain on the numeric pain rating scale (NPRS) had to be greater than 30 mm, the complaints had to be ongoing for more than three months, and the symptoms had to be persistent [6]. 

The following exclusion criteria were applied: age less than 18 years, the absence of first molars, removable dentures, poor oral hygiene, periodontal problems, orofacial pathology unrelated to the TMD diagnosis, acute pain (pain present less than three months), history of head and neck trauma, headache unrelated to TMD (International Classification of Headache Disorders (ICDH II)), pain caused by fibromyalgia, systemic diseases, and diagnosed psychiatric disorders. Participants with pain-free joint clicking and crepitation were also excluded from the study.

The control group (CTR) included 85 healthy volunteers (62 females and 23 males), students and employees from the University of Zagreb’s School of Dental Medicine, and TMD-free patients from the Department of Dentistry at the Clinical Hospital Centre Zagreb. All the participants were above the age of 18 and in generally excellent health. The inclusion criteria for the control group were no history of orofacial pain/discomfort or TMD. 

### 2.1. Diagnosis of Temporomandibular Disorders—Clinical Examination 

Participants were assessed by qualified and experienced physicians (I.Z.A., E.V., M.Z.), who used the Croatian version of the DC/TMD protocol [27]. The examination comprised the palpation of the masticatory muscles and TMJ, the evaluation of lower jaw movements, and the assessment of the presence and nature of TMJ sounds. To establish a definitive diagnosis of TMDp, a patient must confirm the existence of pain in the TMJ and/or masticatory muscles and pain modification (i.e., aggravation or alleviation) during movement, function, or parafunction. Furthermore, during clinical examination, a patient was required to confirm the place and site of the discomfort when triggered by the examiner’s palpation or functional movements of the jaw. To successfully identify the primary problem of a patient, the discomfort felt by the patient had to be labelled as “familiar.” The final diagnosis was determined according to the DC/TMD’s diagnostic decision tree [6].

### 2.2. Assessment of Psychosocial and Psychosomatic Characteristics

All participants, both TMDp patients and control subjects, were asked to complete questionnaires that were part of a self-report instrument set of the DC/TMD protocol (Axis II). The instruments used to assess the psychological status of the participants were Generalised Anxiety Disorder-7 (GAD-7) for anxiety and Patient Health Questionnaire-9 (PHQ-9) for depression. 

Additionally, for assessing psychosomatic characteristics, the participants were asked to fill in two additional questionnaires: the Somatosensory Amplification Scale (SSAS) for assessing somatisation and the Brief Hypervigilance Scale (BHS) for assessing hypervigilance.

The Patient Health Care Questionnaire-9, a 9-item instrument, is designed to screen for depressive symptoms. The Generalised Anxiety Disorder-7, a 7-item questionnaire, is used to assess the severity of anxiety symptoms. Both were based on a four-point Likert scale ranging from “0” (not at all) to “3” (almost every day). The possible outcomes for PHQ-9 ranged from 0 to 27, while GAD-7 values ranged from 0 to 21 [28,29]. 

The Somatosensory Amplification Scale is a 10-item questionnaire designed to examine the proclivity to notice somatic and visceral sensations and perceive them as disproportionately intense. It had ten items with responses ranging from “0” (never) to “4” (always) on a Likert scale and scores ranging from 0 to 40 [30].

The Brief Hypervigilance Scale is a tool used to assess the state of increased vigilance and arousal that can occur in response to perceived threats or stressors. It is a self-report five-item questionnaire, each rated on a five-point Likert scale ranging from “0” (not at all like me) to “4” (very much like me), with scores ranging from 0 to 20 [31].

### 2.3. Assessment of Oral Behavioural Habits

The Oral Behaviours Checklist (OBC), a part of the DC/TMD protocol, was used for identifying and quantifying the frequency of jaw overuse behaviour. It comprises 21 items in total. Each item is scored on a scale from 0 to 4, resulting in a total score range from 0 to 84. However, OBC can be separated into two categories: sleep-related oral behaviours and waking-state oral behaviours. 

Sleep-related oral behaviours consist of two items: (1) teeth grinding and clenching during sleep and (2) potentially harmful sleeping positions for the masticatory system. Both items require information on the frequency of the specific behaviour, resulting in a score of 0 to 8 points. 

The other 19 items focus on oral behaviours during wakefulness (waking-state oral behaviours) that could negatively affect the masticatory system, including activities involving the teeth, tongue, lips, throat, and jaw. Each item requires information on the frequency of the specific behaviour during the day over the preceding month (ranging from “none” to “all of the time”), leading to a score of 0 to 76 points [32]. 

### 2.4. Participant Groups

The study participants were grouped according to the presence of pain into a pain-related TMD (TMDp, *n* = 85) group and a healthy control group (CTR; *n* = 85).

Additional grouping was carried out with respect to OBC scores. Participants were divided into two groups, according to Ohrbach and Knibbe: Diagnostic Criteria for Temporomandibular Disorders (DC/TMD) Scoring Manual for Self-Report Instruments (ACTA): a “high-frequency parafunction” (HFP; *n* = 98) group with OBC sum score 25–84 and a “low-frequency parafunction” (LFP; *n* = 72) group with OBC sum score 1–24.

### 2.5. Extraction of DNA and Genotyping

Following a clinical examination, each subject had a buccal swab obtained with a soft nylon bristle brush (the Cytology Rambrush (Mirandola, Italy, Rimos)). Buccal swab samples were deposited in phosphate-buffered saline (PBS)-filled microtubes (Eppendorf, Hamburg, Germany) and kept at −20 °C for future DNA extraction. The commercially available QIAamp^®^ DNA Mini Kit (QIAGENTM, Venlo, The Netherlands) was used to extract genomic DNA, according to the manufacturer’s instructions. The extracted DNA quality, which was excellent in all samples, was evaluated using 1% agarose gel electrophoresis and ethidium bromide staining. The final DNA concentration was determined by measuring the absorbance at 260 and 280 nm using a NanoDrop™ spectrophotometer (Thermo Fisher Scientific, Waltham, MA, USA) [33].

A total of four SNPs in genes encoding enzymes with antioxidative properties (*SOD2* (rs4880), *CAT* (rs1001179), *GPX1* (rs1050450), and *NQO1* (rs689452) (Gene IDs can be seen in Appendix A)) were genotyped using predeveloped Taqman™ assays and a TaqPath™ ProAmp™ Master Mix (Thermo Fisher Scientific, Waltham, MA, USA) on an ABI 7300 Real-Time PCR Instrument System (Applied Biosystems, Waltham, MA, USA), according to manufacturer’s instructions. In each experiment, positive controls encompassing all conceivable genotypes and “no template” controls were used.

### 2.6. Selection of SNPs

Glutathione peroxidase 1 (*GPX1*) polymorphism (rs1050450) results in the occurrence of nucleotides C/T at the position 599 (reference GPX1 transcript variant 1 Primary Assembly NM_000581.4: C599 > T599), resulting in the replacement of proline with leucine (CCC > CTC; Pro200Leu). It is proposed that enzymes with leucine in their protein structure have decreased enzymatic activity [34].

Superoxide dismutase 2 (*SOD2*) polymorphism (rs4880) results in the occurrence of nucleotides T/C at position 47 (reference SOD2 transcript variant 1 primary assembly NM_000636.4: T47 > C47), resulting in the replacement of valine with alanine (GTT > GCT; Val16Ala). It is proposed that valine decreases enzyme activity and leads to increased oxidative stress [35].

Catalase (*CAT*) polymorphism (rs1001179) results in the occurrence of nucleotides C/T at position 1317 (reference GRCh38.p14 primary assembly NM_001752.4: C1317 > T1317), which is −250 bp upstream from the start codon.

NAD(P)H quinone dehydrogenase 1 (*NQO1*) polymorphism (rs689452) results in the occurrence of nucleotides C/G at the position 69718561 (reference GRCh38.p14 primary assembly NC_000016.10g (69709401.69726560): C69718561 > G69718561; IVS1-27C > G), in the first *NQO1* intron. 

No studies have investigated the involvement of these SNPs with the occurrence of TMD and harmful oral behaviours. However, it is possible that the presence of these SNPs could impact the antioxidative stress response, which is possibly involved in the aetiology of chronic pain disorders.

### 2.7. Statistical Analysis

IBM SPSS Statistics for Windows, Version 26.0 (Armonk, NY, USA: IBM Corp) was used for statistical analysis. 

In a case–control study, for the dominant inheritance model with a 1:1 participant ratio, a sample size of 150 patients (75 control participants and 75 TMD patients) is required for achieving a power of 80% with a statistical error of 5% (α  =  0.05). This calculation was based on the assumption of a 5% TMD prevalence rate, as determined by previous studies [36,37].

Chi-squared tests were used for assessing deviations from the Hardy–Weinberg equilibrium. Differences between the studied groups (TMDp vs. CTR) and (HFP vs. LFP) were tested using the Mann–Whitney U test and chi-squared test, for psychological and psychosomatic characteristics and categorical variables, respectively.

Genotype frequency distribution among the groups (TMDp and CTR; HFP and LFP) was compared using the chi-squared test and Fisher’s exact test. The assessment was performed according to dominant and recessive genetic models. In both models, the minor allele represented the risk allele.

The Mann–Whitney U test was used to examine the differences in psychological and psychosomatic characteristics with respect to specific genotypes, for each selected SNP. 

To assess the associations of genetic, psychological, and psychosomatic parameters with pain-related TMD and with OB frequency, Spearman’s correlation coefficients were used. 

Multiple logistic regression analysis was used for revealing the factors associated with pain-related TMD. Finally, multiple linear regression models were developed to clarify the effects of various factors related to OB frequency and severity. In multiple linear regression models, the predictors of oral behaviour frequency were analysed when controlling for demographic variables (age and sex) and the presence of pain-related TMD (myalgia/arthralgia or absence of TMD). The independent variables were the examined genotype, depression, anxiety, somatosensory amplification, and hypervigilance. Variables significantly associated with a dependent variable were entered into the linear regression models. *p* values less than 0.05 were considered statistically significant.

## 3. Results

### Characteristics of Participants

Table 1 shows the sociodemographic characteristics and other study-relevant characteristics of the participants. The TMDp group consisted of 85 patients, 76 of whom were women. The control group included 85 participants, 62 of whom were women. Women were more represented in the TMPp than the CTR group (89.4% vs. 72.9%, *p* = 0.006). No significant differences in other general characteristics between the two groups were found (age: *p* = 0.064; educational level: *p* = 0.676). Trait anxiety, depression, hypervigilance, and somatosensory amplification did not differ between TMDp patients and the healthy controls (*p* = 0.427, *p* = 0.329, *p* = 0.362, *p* = 0.998, respectively). Table 2 presents the frequency of oral behaviours in TMDp patients and the CTR group. Significantly higher values were found for sleep-related oral behaviours (5.21 ± 2.23 vs. 4.34 ± 1.76, *p* = 0.007), while OBC-tot score and waking-state oral behaviours did not differ significantly between these two groups (29.05 ± 10.75 vs. 25.92 ± 7.26, *p* = 0.161; 24.09 ±10.12 vs. 21.63 ± 6.85, *p* = 0.235, respectively).

Based on the OBC-tot score results, 57.6% (*n* = 98) of the total number of participants were identified as having high-frequency parafunction (48 of whom belonged to the TMDp and 50 to the CTR group). The rest of the participants, 72 of them, were identified as having low-frequency parafunction. When comparing HFP with LFP individuals (Table 1), women were more represented in the HFP group (86.7% vs. 73.6%, *p* = 0.031), while no significant differences were found for age (*p* = 0.077) and education level (*p* = 0.804). HFP individuals presented significantly higher anxiety (5.05  ±  4.22 vs. 3.42  ±  2.77, *p* =  0.020) and depression scores (6.01  ±  4.68 vs. 3.90  ±  3.30, *p* =  0.001), as well as higher hypervigilance (4.59 ± 3.48 vs. 3.47 ± 2.89, *p* = 0.046) and somatosensory amplification (15.79 ± 5.85 vs. 12.19 ± 5.03, *p* < 0.001) than LFP individuals.

## 4. Participants’ Genotype

The genotype distribution of the four SNPs, with respect to the presence of pain (TMDp/CTR) and frequency of oral behaviours (HFP/LFP), are presented in Table 3 and Table 4 for recessive and dominant models, respectively.

Our analysis of the recessive model (Table 3) revealed that there was no difference in the distribution of rs1001179, rs4880, and rs1050450 genotypes when TMDp patients were compared with the healthy controls (*p* = 0.773, *p* = 0.701, *p* = 0.201, respectively). When assessing the effect of oral behaviour frequency, a significant difference was found in genotype distribution for rs1050450 (*GPX1*) between HFP and LFP individuals; the frequency of rs1050450 AA genotype was higher in the HFP group than in the LFP group (14.3% vs. 4.2%, *p*  = 0.030). The frequency of patients carrying the minor allele T of rs1001179, as well as the frequency of patients carrying the minor allele G of rs4880, was also slightly higher in the HFP group than in the LFP group, but the difference was not statistically significant.

There was no difference in the distribution of rs1001179, rs4880, rs1050450, and rs689452 genotypes between TMDp and CTR, nor between HFP and LFP individuals, when the dominant model was analysed (Table 4).

## 5. Psychological and Psychosomatic Traits with Respect to a Specific Genotype

Data regarding the differences in psychological, psychosomatic, and behavioural characteristics for the *CAT*, *SOD2*, *GPX*1, and *NQO1* genes according to the respective genotype group are available on request. Only characteristics that were significantly related to a specific genotype are presented in Figures that follow. 

### 5.1. Analysis with Respect to the Presence of Pain (TMDp/CTR)

**Figure 1:**
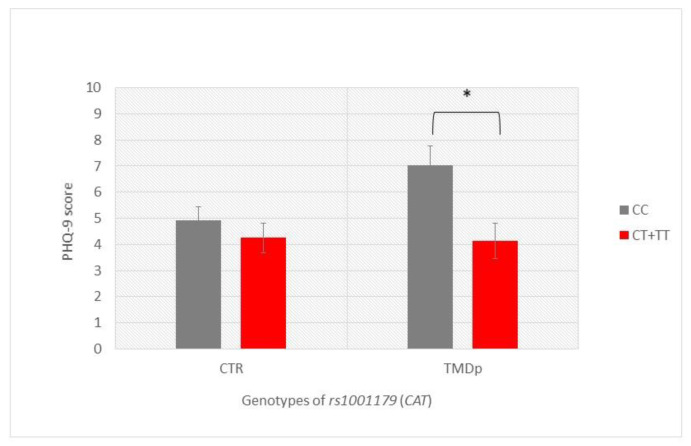
Patient Health Questionnaire-9 scores of participants with different genotypes of rs1001179 (*CAT*), presenting a comparison of patients with pain-related TMD and control subjects. Data are expressed as mean ± SME; * represents *p* < 0.05. Abbreviations: CTR, control group—absence of TMD; TMDp, TMD-pain patients—presence of pain disorders, including myalgia, arthralgia, or both; PHQ-9, Patient Health Questionnaire-9.

Our analysis of the examined psychological and psychosomatic characteristics according to genotype revealed that TMDp patients, CC *CAT* (rs1001179) carriers, reported significantly higher depression scores than the CT + TT genotype carriers (PHQ-9: 7.1 vs. 4.2, *p* = 0.002). In the control group, the examined characteristics were not related to a specific genotype (Figure 1). 

**Figure 2:**
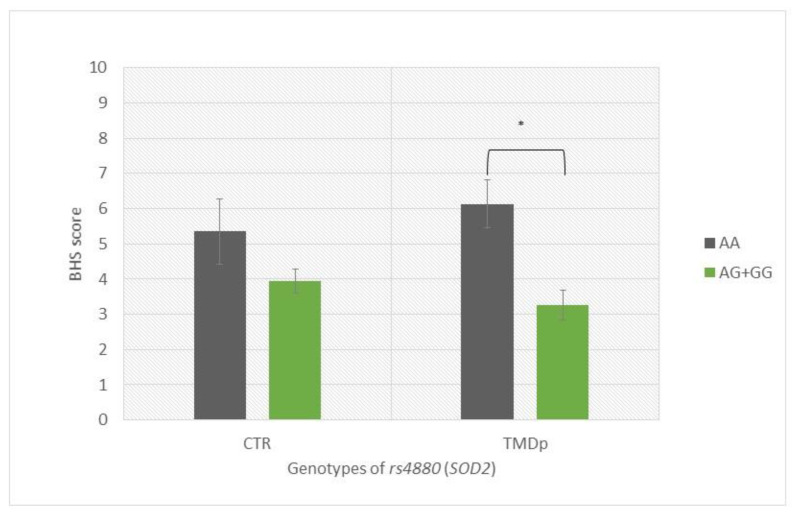
Brief Hypervigilance Scale (BHS) scores of participants with different genotypes of rs4880 (SOD2), presenting a comparison of patients with pain-related TMD and control subjects. Data are expressed as mean ± SME; * represents *p* < 0.05. Abbreviations: CTR, control group—absence of TMD; TMDp, TMD-pain patients—presence of pain disorders, including myalgia, arthralgia, or both; BHS, Brief Hypervigilance Scale.

TMDp patients, AA *SOD2* (rs4880) homozygotes, reported significantly higher hypervigilance scores than TMDp patients carrying the AG + GG genotypes (BHS: 6.14 vs. 3.25, *p* = 0.0001) (Figure 2). 

**Figure 3:**
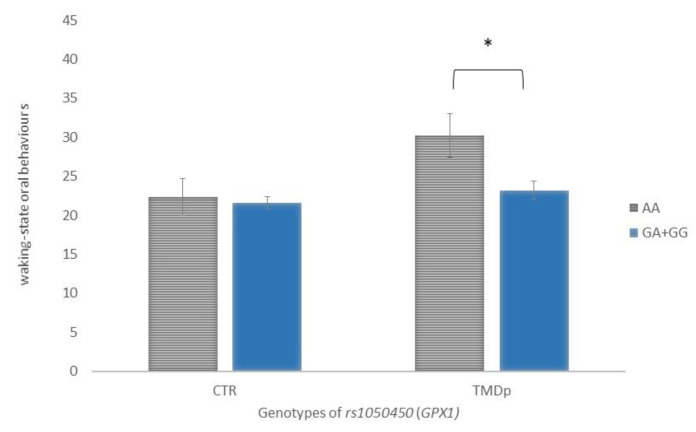
Waking-state oral behaviour scores of participants with different genotypes of rs1050450 *(GPX1)*, presenting a comparison of patients with pain-related TMD and control subjects. Data are expressed as mean ± SME; * represents *p* < 0.05. Abbreviations: CTR, control group—absence of TMD; TMDp, TMD-pain patients—presence of pain disorders, including myalgia, arthralgia, or both.

When behavioural characteristics were analysed according to genotype, it was found that TMDp patients, homozygous for the minor allele A of the rs1050450 *GPX1,* reported significantly more waking-state OBs than the GA + GG genotype carriers (score: 30 vs. 23, *p* = 0.019). In the control group, waking-state OBs were not associated with examined genotype (Figure 3).

### 5.2. Analysis with Respect to Oral Behaviour Frequency (HFP/LFP)

**Figure 4:**
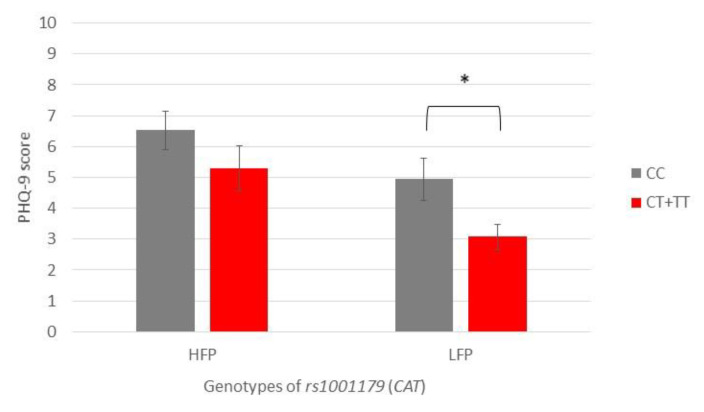
Patient Health Questionnaire-9 scores of participants with different genotypes of rs1001179 *(CAT)*, showing a comparison of participants with high- and low-frequency parafunction. Data are expressed as mean ± SME; * represents *p* < 0.05. Abbreviations: HFP, high-frequency parafunction group; LFP, low-frequency parafunction group; PHQ-9, Patient Health Questionnaire-9.

Subjects carrying the CC genotype of *CAT* rs1001179 polymorphism reported higher depression scores than CT + TT genotype carriers, with significant differences present only in the LFP group (PHQ-9: 4.9 vs. 3.0, *p* = 0.021) (Figure 4). 

**Figure 5:**
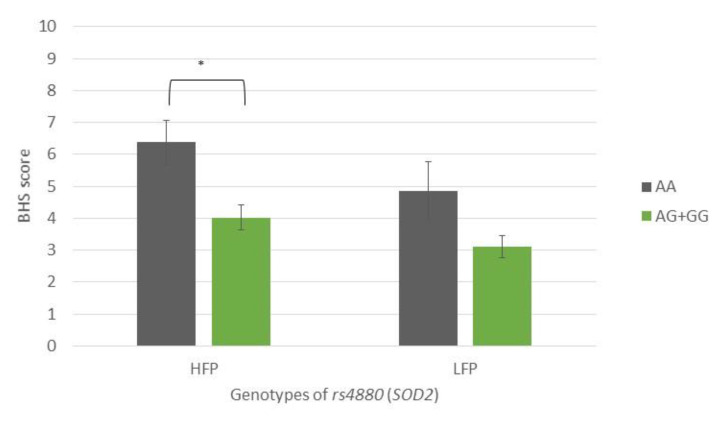
Brief Hypervigilance Scale (BHS) scores of participants with different genotypes of rs4880 (*SOD2*), showing a comparison of participants with high- and low-frequency parafunction. Data are expressed as mean ±SME; * represents *p* < 0.05. Abbreviations: HFP, high-frequency parafunction group; LFP, low-frequency parafunction group; BHS, Brief Hypervigilance Scale.

HFP subjects with the AA genotype of the *SOD2* SNP rs4880 reported significantly higher hypervigilance scores than AG + GG genotype carriers (BHS: 6.4 vs. 4.0, *p* = 0.003) (Figure 5). 

## 6. Risk Factors Associated with Pain-Related TMD 

In order to find the factors associated with pain-related TMD, a multivariable logistic regression model was used. Age-, sex-, and sleep-related OBs were included as potential confounders or effect modifiers. The probability of TMDp was significantly associated with a higher frequency of sleep-related OBs (OR 1.198, 95% CI 1.020–1.406, *p* = 0.028), female sex (OR 3.190, 95% CI 1.329–7.654, *p* = 0.009), and age increase (OR 1.041, 95% CI 1.005–1.078, *p* = 0.025) (Table 5). The model explains roughly 15% of the variation in the outcome (Nagelkerke R^2^).

## 7. Risk Factors Associated with Oral Behaviour Frequency

### 7.1. Risk Factors Associated with Waking-State Oral Behaviours

The AA genotype of rs1050450 *(GPX1)* as well as depression, anxiety, somatosensory amplification, hypervigilance, and sex were found to have a significant association with waking-state oral behaviours. Due to the strong correlation between depression, anxiety, somatosensory amplification, and hypervigilance, these variables were entered into the model independently (Table 6). 

The multiple linear regression analysis revealed that, after adjustment for age, a significant predictor for waking-state oral behaviours was depression, followed by female sex and AA genotype of rs1050450. The whole regression model accounted for 18.1% of the variance (R^2^ = 0.181).

When substituted for depression, an increase in anxiety by one scalar point increased waking-state oral behaviour score by 0.721 scalar points, while female sex and *GPX1* polymorphism remained significant. The whole regression model accounted for 15.0% of the variance (R^2^ = 0.150). 

One-point increment in the somatosensory amplification score was associated with 0.604 points increments in the waking-state oral behaviour score, while an increase in hypervigilance by one scalar point increased the waking-state oral behaviour score by 0.619 scalar points. However, the *GPX1* polymorphism rs1050450 did not remain significant in these models.

### 7.2. Risk Factors Associated with Sleep-Related Oral Behaviours 

Age, pain-related TMD, depression, anxiety, and somatosensory amplification were shown to have a significant association with sleep-related oral behaviours. The multiple linear regression analysis (Table 7) revealed that, after adjustment for sex, a significant predictor for sleep-related OBs was depression. Furthermore, the presence of TMDp increased the sleep-related oral behaviour scores by 0.636 scalar points. The whole regression model accounted for 7.4 % of the variance (R^2^ = 0.074). 

Due to the high correlation with depression, anxiety (r = 0.676; *p* < 0.001) and somatosensory amplification (*r* = 0.354; *p* < 0.001) were first omitted from the analysis. When substituted for depression, both anxiety and somatosensory amplification showed a significant effect on sleep-related oral behaviour scores. 

## 8. Discussion

In the present study, we examined the relationship between pain-related TMD, oral behaviours, psychological and psychosomatic symptoms, and selected SNPs in four genes encoding for proteins with antioxidative properties. 

To begin the discussion of our findings, it is important to offer a brief review of the general TMD-related characteristics of the participants. The participants with a high frequency of oral parafunction, regardless of pain status, had significantly higher scores of somatosensory amplification, anxiety, depression, and hypervigilance. These results might suggest that individuals who engage in more frequent oral parafunction may be at a higher risk of developing these psychological symptoms, or those with psychological symptoms may be at a higher risk of developing a high frequency of oral parafunction.

Interestingly, no significant differences were observed in the levels of somatosensory amplification, anxiety, depression, or hypervigilance between TMDp patients and the healthy controls. This implies that psychological symptoms may be more closely related to parafunctional behaviours than TMD.

We found that TMDp patients had a higher frequency of sleep-related oral behaviours than the healthy controls. Although there was a difference in waking-state oral behaviours between TMDp patients and the healthy controls, this difference was not statistically significant. Additionally, the frequency of oral parafunction differed significantly between genders, with more women engaging in a high-frequency parafunction and more men engaging in a low-frequency parafunction. These findings indicate that oral parafunctional behaviours may be linked to psychological symptoms, and it is possible that a gender difference needs to be considered when evaluating and treating individuals with these behaviours. However, it is important to bear in mind the overall disparity in the frequency of women versus men in the study, as there were more women included. Additionally, this association remains controversial in the literature, with some studies reporting differences in parafunctional activities between men and women, while others report no such differences [38,39,40]. Additionally, our findings suggest that sleep-related oral behaviours may be particularly relevant in TMD patients. 

Regarding the genotype distribution of polymorphisms in the four oxidative-stress-related genes, no differences were found between TMDp patients and the healthy controls, neither in recessive nor in dominant models. This could mean that, without considering additional risk factors, the inherited variations of the selected SNPs would not be recognised as potential risk factors related to TMD. However, when psychological and psychosomatic characteristics and oral behaviours were evaluated, the examined characteristics were influenced by genotype only in the TMDp group. Specifically, in patients with pain-related TMD, the genotype AA of *SOD2* polymorphism rs4880 was associated with higher hypervigilance scores, while the genotype CC of *CAT* SNP rs1001179 was associated with higher depression scores. In our earlier work, we discovered a link between psychological characteristics in TMD patients and antioxidant status, where depression and anxiety were both negatively correlated with antioxidant levels. Furthermore, the improvement in depressive symptoms during therapy was associated with a decrease in total antioxidant capacity [41]. Such results might be, among other reasons, a reflection of genetic variations found among TMD patients. Both mentioned genotypes contain two copies of the major allele, even though a minor allele was expected to be a risk factor in this study. Some research reported that allele A of rs1001179 (*CAT*) was associated with a 0.6-fold decrease in risk for acoustic neuroma [42]. Galasso et al. [43] revealed the importance of the *CAT* rs1001179 polymorphism with respect to the *CAT* gene activity. In their model of investigation, leukemic cells harbouring the rs1001179 SNP T allele exhibited a significantly higher *CAT* expression than the cells bearing the CC genotype, due to the increased binding of two transcription factors, ETS proto-oncogene 1 (ETS-1) and glucocorticoid receptor beta (GR-β). The strength of binding and transcriptional activity of *CAT* was also dependent on the level of DNA methylation [43]. These data imply that even though minor alleles are more likely to be risk alleles in complex diseases, this may not always be the case [44]. 

Since migraine is a chronic pain disorder sometimes found as a comorbid condition to TMD, it is interesting to see whether there is a potential genetic overlap. Papasavva et al. investigated how variabilities in SNPs, including rs4880, rs1001179, and rs1050450, are related to susceptibility to the clinical phenotypes and features of migraine. What they found is that homozygosity for the minor T allele (rs1001179) was associated with the later age at the onset when compared to homozygosity for the more common allele C [45]. It would be interesting to further investigate the connection between TMD and migraine onset and depression. Considering *SOD2*, no significant association was found for rs4880 variants for clinical features of migraine [45]. One case–control study reported that there was a lack of association between the SNPs of oxidative-stress-related genes (among which was rs4880 of *SOD2*) and chronic migraine [46].

Our findings revealed that polymorphisms in oxidative-stress-related genes were not predictive for pain-related TMD. The recessive homozygous genotype of the *GPX1* polymorphism rs1050450, together with greater anxiety and depression scores and the female gender, was revealed to be a significant predictor of more frequent waking-state oral behaviours. Sleep-related oral behaviours were not predicted by any of the examined gene polymorphisms. Rather, they were predicted by increased age, greater anxiety, depression, and somatisation scores. These findings suggest that oxidative-stress-related genes may play a role in waking-state oral behaviours, while sleep-related parafunctional activity and pain-related TMD is more likely consequences of the complex interplay of various biological and psychosocial factors. 

Glutathione peroxidase 1 is a critical endogenous antioxidant. This enzyme is encoded by the *GPX1* gene. Its polymorphism, rs1050450, contains nucleotides C/T in exon 2, resulting in an amino acid substitution of proline to leucine [47,48]. The anticodon-biding assay was employed in our study, which indicates that the “AA” genotype may be read as “TT” and “GG” as “CC.” With this in mind, we may discuss the scientific and clinical context of our findings. Rs1050450 is linked to altered enzyme activity and may have an impact on an individual’s antioxidant capability. The minor T allele has been linked to lower *GPX1* activity [49]. This polymorphism has been linked to a number of diseases, including peripheral neuropathy, cardiovascular illness, and migraine [45,50,51]. The genotype containing two copies of the T allele was associated with patients who experienced longer migraine episodes than patients who had shorter migraine attacks, indicating that the presence of the T allele appears to be connected to longer migraine attacks [45]. We found that the individuals carrying genotype TT of rs1050450 are at a higher risk for experiencing high-frequency waking-state parafunction. Parafunction during wakefulness is more closely linked to various manifestations of stress, which might also be reflected through the variability in antioxidant defence enzyme activities [52].

Our study has several limitations that need to be taken into account when interpreting the data. One of these limitations is the potential bias arising from the fact that in CTR and TMDp groups, the participants were not sex-matched. This problem was addressed by applying additional analyses to determine if the odds ratios would remain significant when accounting for relevant risk variables and covariates such as sex. Since there could be sex-related differences in pain physiology and clinical outcomes, these additional analyses were critical. Additionally, our control group represents the general population, in contrast to TMD-prone patients who were predominantly women. Even though our sample size was suitable and adequate according to power analysis, studies with larger sample sizes would yield more valuable insights and produce more conclusive results. Lastly, despite the fact that most of the questionnaires used in this study have been validated and are part of the recommended DC/TMD protocol for research studies, it is important to consider the possibility of participants not providing truthful responses in self-reported questionnaires, especially on sensitive questions. All the participants filled out questionnaires in the same clinical setting with a certain level of privacy.

Overall, these findings highlight the need for a comprehensive approach to diagnosing and treating TMD. While genetic factors may play a role to some extent, it is still important to consider a range of other factors such as age, sex, and psychological characteristics when assessing and managing TMD and their associated sleep-related oral behaviours. The interindividual variability in the distribution of oxidative-stress-related genes’ polymorphisms may not have a direct association with the development of pain-related TMD. However, they may play an important role in the complex interaction between TMD, behavioural habits, and psychological characteristics of a patient. 

The role of the studied SNPs remains largely unexplored in the field of chronic pain and TMD, and further studies on this topic are needed. 

## 9. Conclusions

Based on the results of this study, it can be concluded that TMDp cannot be directly linked to described polymorphisms in the four selected oxidative-stress-related genes. However, TMDp patients, homozygous for the minor allele A of rs1050450 *GPX1*, reported significantly more waking-state oral behaviours than the GA + GG genotype carriers. This finding was not observed in the healthy participants. 

We found that the homozygous AA carriers of the rs1050450 SNP were more often represented in the HFP group than among the LFP participants. This genotype proved to be a significant risk factor for increased frequency of waking-state oral behaviours, while sleep-related oral behaviours were not shown to be associated with the investigated genetic factors.

The association of waking-state oral behaviours with the polymorphisms of oxidative-stress-related genes additionally supports previous observations that daytime bruxism is more closely linked to various manifestations of stress, which might also be reflected through the variability in the antioxidant defence enzyme activities.

## Figures and Tables

**Table 1:** Characteristics of study participants.

Variable			Pain Presence		Oral Behaviours Frequency	
			CTR (*n* = 85)	TMDp (*n* = 85)	LFP (*n* = 72)	HFP (*n* = 98)
Gender	Female, *n* (%)		62 (72.9%)	76 (89.4%)	53 (73.6%)	85 (86.7%)
	Male, *n* (%)		23 (27.1%)	9 (10.6%)	19 (26.4%)	13 (13.3%)
	*p* ^b^		**0.006**		**0.031**	
Age	Female	Mean (SD)	26.15 (7.71)	29.51 (11.01)	29.51 (10.45)	27.06 (9.27)
		*p* ^a^	0.067		0.083	
	Male	Mean (SD)	26.52 (7.91)	33.78 (12.22)	30.63 (10.96)	25.54 (6.79)
		*p* ^a^	0.064		0.077	
Education level	Elementary school, *n* (%)		/	6 (7%)	1 (1.4%)	5 (5.1%)
	High school, *n* (%)		/	24 (28%)	15 (20.8%)	9 (9.2%)
	Student, *n* (%)		58 (68%)	23 (27%)	31 (43.1%)	50 (51%)
	College Degree, *n* (%)		20 (24%)	25 (30%)	18 (25%)	27 (27.6%)
	Master’s Degree, *n* (%)		7 (8%)	7 (8%)	7 (9.7/%)	7 (7.1%)
	*p* ^b^		0.676		0.804	
Somatosensory amplification (SSAS) (0–40)	Mean (SD)		14.09 (5.07)	14.43 (6.44)	12.19 (5.03)	15.79 (5.85)
	*p* ^a^		0.998		**<0.001**	
Anxiety symptom severity (GAD-7) (0–21)	**Mean (SD)**		4.06 (3.49)	4.65 (3.99)	3.42 (2.77)	5.05 (4.22)
	*p* ^a^		0.427		**0.020**	
Hypervigilance (BHS) (0–20)	Mean (SD)		4.24 (3.08)	4.00 (3.49)	3.47 (2.89)	4.59 (3.48)
	*p* ^a^		0.362		**0.046**	
Depression symptom severity (PHQ-9) (0–27)	Mean (SD)		4.60 (3.59)	5.63 (4.82)	3.90 (3.30)	6.01 (4.68)
	*p* ^a^		0.329		**0.001**	

Abbreviations: CTR, control group—absence of TMD; TMDp, TMD-pain patients—presence of pain disorders including myalgia, arthralgia, or both; HFP, high-frequency parafunction group; LFP, low-frequency parafunction group; GAD-7, Generalized Anxiety Disorder-7; PHQ-9, Patient Health Questionnaire-9; SSAS, Somatosensory Amplification Scale; BHS, Brief Hypervigilance Scale; *n*, number of participants; *p*, p-value; SD, standard deviation. Significant values are displayed in bold. ^a^ Mann–Whitney *U* test. ^b^ Chi-squared test.

**Table 2:** Description of the patients and controls based on oral behaviour frequency.

Variable		CTR (*n* = 85)	TMDp (*n* = 85)
Oral Behaviours Checklist (OBC) total score (0–84)	Mean (SD)	25.92 (7.26)	29.05 (10.75)
	*p* ^a^	0.161	
.Sleep-related oral behaviours (0–8)	Mean (SD)	4.34 (1.76)	5.21 (2.23)
	*p* ^a^	**0.007**	
Waking-state oral behaviours (0–76)	Mean (SD)	21.63 (6.85)	24.09 (10.12)
	*p* ^a^	0.235	

*n*, number of participants; *p*, *p*-value; SD, standard deviation. Significant values are displayed in bold. ^a^ Mann–Whitney *U* test.

**Table 3:** Distribution of genotypes with respect to pain presence (TMDp patients vs. controls) and frequency of oral behaviours (low-frequency parafunction vs. high-frequency parafunction)—recessive model.

	TMDp (*n* = 85)		CTR (*n* = 85)		LFP (*n* = 72)		HFP (*n* = 98)	
rs1001179 *(CAT)* *n (%)* *p*	TT	CT + CC	TT	CT + CC	TT	CT + CC	TT	CT + CC
	6 (7.1%)	79 (92.9%)	7 (8.2%)	78 (91.8%)	4 (5.6%)	68 (94.4%)	9 (9.2%)	89 (90.8%)
	0.773				0.379			
rs4880 *(SOD2)* *n (%)* *p*	GG	AG + AA	GG	AG + AA	GG	AG + AA	GG	AG + AA
	16 (18.8%)	69 (81.2%)	18 (21.2%)	67 (78.8%)	14 (19.4%)	58 (80.6%)	20 (20.4%)	78 (79.6%)
	0.701				0.877			
rs1050450 *(GPX1)* *n (%)* *p*	AA	GA + GG	AA	GA + GG	AA	GA + GG	AA	GA + GG
	11 (12.9%)	74 (87.1%)	6 (7.1%)	79 (92.9%)	3 (4.2%)	69 (95.8%)	14 (14.3%)	84 (85.7%)
	0.201				**0.030**			
rs689452 *(NQO1)* *n (%)* *p*	CC	CG + GG	CC	CG + GG	CC	CG + GG	CC	CG + GG
	0 (0%)	85 (100%)	0 (0%)	85 (100%)	0 (0%)	72 (100%)	0 (0%)	98 (100%)
	^a^				^a^			

Abbreviations: CTR, control group—absence of TMD; TMDp, TMD-pain patients—presence of pain disorders, including myalgia, arthralgia, or both; HFP, high-frequency parafunction group; LFP, low-frequency parafunction group; *n*, number of participants; *p*, *p*-value; CAT, catalase; SOD2, super oxide dismutase 2; GPX1, glutathion peroxidase 2; NQO1, NAD(P)H quinone dehydrogenase 1. Significant values are displayed in bold. ^a^ It was not possible to perform analysis due to the lack of participants carrying both minor alleles.

**Table 4:** Distribution of genotypes with respect to pain presence (TMDp patients vs. controls) and frequency of oral behaviours (low-frequency parafunction vs. high-frequency parafunction)—dominant model.

	TMDp *(n* = 85)		CTR (*n* = 85)		LFP (*n* = 72)		HFP (*n* = 98)	
rs1001179 *(CAT)* *n (%)* *p*	CC	CT + TT	CC	CT + TT	CC	CT + TT	CC	CT + TT
	44 (51.8%)	41 (48.2%)	45 (52.9%)	40 (47.1%)	32 (44.4%)	40 (55.6%)	57 (58.2%)	41 (41.8%)
	0.878				0.077			
rs4880 *(SOD2)* *n (%)* *p*	AA	AG + GG	AA	AG + GG	AA	AG + GG	AA	AG + GG
	22 (25.9%)	63 (74.1%)	17 (20%)	68 (80%)	15 (20.8%)	57 (79.2%)	24 (24.5%)	74 (75.5%)
	0.362				0.575			
rs1050450 *(GPX1)* *n (%)* *p*	GG	GA + AA	GG	GA + AA	GG	GA + AA	GG	GA + AA
	42 (49.4%)	43 (50.6%)	35 (41.2%)	50 (58.8%)	35 (48.6%)	37 (51.4%)	42 (42.9%)	56 (57.1%)
	0.281				0.456			
rs689452 *(NQO1)* *n (%)* *p*	GG	CG + CC	GG	CG + CC	GG	CG + CC	GG	CG + CC
	63 (74.1%)	22 (25.9%)	66 (77.6%)	19 (22.4)	56 (77.8%)	16 (22.2%)	73 (74.5%)	25 (25.5%)
	0.591				0.620			

Abbreviations: CTR, control group—absence of TMD; TMDp, TMD-pain patients—presence of pain disorders, including myalgia, arthralgia, or both; HFP, high-frequency parafunction group; LFP, low-frequency parafunction group; *n*, number of participants; *p*, p-value; CAT, catalase; SOD2, super oxide dismutase 2; GPX1, glutathion peroxidase 2; NQO1, NAD(P)H quinone dehydrogenase 1.

**Table 5:** Multiple logistic regression results for predictors of pain-related TMD.

	B	SE	*p*	OR	95% CI
Sleep-related oral behaviours	0.180	0.082	0.028 *	1.198	1.020–1.406
Sex (male_0; female_1)	1.160	0.447	0.009 *	3.190	1.329–7.654
Age	0.040	0.018	0.025 *	1.041	1.005–1.078

B, no standardised variable coefficient; SE, standard error; * statistically significant; OR, odds ratio; CI, confidence interval. Significant values are displayed in bold.

**Table 6:** Multiple linear regression results for predicting the effect of each variable on waking-state oral behaviours’ frequency.

	B	SE	β	*p*
rs1050450 (*GPX1*) (Heterozygous GA + GG_0; -homozygous AA_1)	1.370	0.683	0.142	0.047 *
PHQ-9 score	0.724	0.142	0.356	<0.001 *
Sex (male_0; female_1)	3.934	1.555	0.177	0.012 *
Age	−0.110	0.063	−0.123	0.083
rs1050450 (*GPX1*) (Heterozygous GA + GG_0; -homozygous AA_1)	1.422	0.696	0.148	0.043 *
GAD-7 score	0.721	0.165	0.311	<0.001 *
Sex (male_0; female_1)	3.331	1.590	0.150	0.038 *
Age	−0.087	0.064	−0.097	0.177
rs1050450 (*GPX1*) (Heterozygous GA + GG_0; -homozygous AA_1)	1.166	0.670	0.121	0.084
Somatosensory amplification score	0.604	0.104	0.402	<0.001 *
Sex (male_0; female_1)	2.858	1.534	0.129	0.064
Age	−0.098	0.062	−0.110	0.114
rs1050450 (*GPX1*) (Heterozygous GA + GG_0; -homozygous AA_1)	1.290	0.714	0.134	0.073
Hypervigilance score	0.619	0.196	0.234	0.002 *
Sex (male_0; female_1)	4.076	1.625	0.184	0.013 *
Age	−0.061	0.067	−0.068	0.362

* Statistically significant; B, no standardised variable coefficient; SE, standard error; **β**, standardised variable coefficient. Significant values are displayed in bold.

**Table 7:** Multiple linear regression results for predicting the effect of each variable on sleep-related oral behaviours’ frequency.

	B	SE	β	*p*
PHQ-9 score	0.091	0.036	0.189	0.012 *
TMDp (no_0; yes_1)	0.636	0.318	0.155	0.048 *
Sex (male_0; female_1)	0.299	0.379	0.057	0.452
Age	0.025	0.016	0.117	0.123
GAD-7 score	0.089	0.041	0.163	0.030 *
TMDp (no_0; yes_1)	0.681	0.319	0.167	0.034 *
Sex (male_0; female_1)	0.214	0.400	0.041	0.594
Age	0.027	0.016	0.128	0.094
Somatosensory amplification score	0.055	0.027	0.156	0.040 *
TMDp (no_0; yes_1)	0.728	0.318	0.178	0.023 *
Sex (male_0; female_1)	0.171	0.403	0.033	0.671
Age	0.026	0.016	0.121	0.112

* Statistically significant. B, no standardised variable coefficient. SE, standard error. **β**, standardised variable coefficient. Significant values are displayed in bold.

## Data Availability

Data is contained within the article and supplementary material. Any supplementary files that may be relevant to this research are available from the corresponding author upon reasonable request.

## Supplementary Materials

The following supporting information can be downloaded at: https://www.mdpi.com/article/10.3390/antiox12061195/s1, Table S1, STROBE checklist.

## Appendix A

The genes that were used in this study all had NCBI gene IDs: *GPX1* (rs1050450)—2876; *SOD2* (rs4880)—6648; *CAT* (rs1001179)—847; *NQO1* (rs689452)—1728.

## References

[1] Valesan L.F., Da-Cas C.D., Réus J.C., Denardin A.C.S., Garanhani R.R., Bonotto D., Januzzi E., de Souza B.D.M. (2021). Prevalence of temporomandibular joint disorders: A systematic review and meta-analysis. Clin. Oral Investig..

[2] Maixner W., Diatchenko L., Dubner R., Fillingim R.B., Greenspan J.D., Knott C., Ohrbach R., Weir B., Slade G.D. (2011). Orofacial pain prospective evaluation and risk assessment study—The OPPERA study. J. Pain.

[3] Slade G.D., Bair E., Greenspan J.D., Dubner R., Fillingim R.B., Diatchenko L., Maixner W., Knott C., Ohrbach R. (2013). Signs and symptoms of first onset TMD and sociodemographic predictors of its development: The OPPERA prospective cohort study. J. Pain.

[4] Adèrn B., Stenvinkel C., Sahlqvist L., Tegelberg Å. (2014). Prevalence of temporomandibular dysfunction and pain in adult general practice patients. Acta Odontol. Scand..

[5] Ferrillo M., Giudice A., Marotta N., Fortunato F., Di Venere D., Ammendolia A., Fiore P., de Sire A. (2022). Pain Management and Rehabilitation for Central Sensitization in Temporomandibular Disorders: A Comprehensive Review. Int. J. Mol. Sci..

[6] Schiffman E., Ohrbach R., Truelove E., Look J., Anderson G., Goulet J.P., List T., Svensson P., Gonzalez Y., Lobbezoo F. (2014). International RDC/TMD Consortium Network, International association for Dental Research; Orofacial Pain Special Interest Group, International Association for the Study of Pain. Diagnostic Criteria for Temporomandibular Disorders (DC/TMD) for Clinical and Research Applications: Recommendations of the International RDC/TMD Consortium Network * and Orofacial Pain Special Interest Group ^†^. J. Oral Facial Pain Headache.

[7] Ohrbach R., Michelotti A. (2018). The Role of Stress in the Etiology of Oral Parafunction and Myofascial Pain. Oral Maxillofac. Surg. Clin. N. Am..

[8] Manfredini D., Ahlberg J., Lobbezoo F. (2022). Bruxism definition: Past, present, and future-What should a prosthodontist know?. J. Prosthet. Dent..

[9] Kara M.I., Yanık S., Keskinruzgar A., Taysi S., Copoglu S., Orkmez M., Nalcaci R. (2012). Oxidative imbalance and anxiety in patients with sleep bruxism. Oral Surg. Oral Med. Oral Pathol. Oral Radiol..

[10] Iodice G., Cimino R., Vollaro S., Lobbezoo F., Michelotti A. (2019). Prevalence of temporomandibular disorder pain, jaw noises and oral behaviours in an adult Italian population sample. J. Oral Rehabil..

[11] Braz M.A., Freitas Portella F., Seehaber K.A., Bavaresco C.S., Rivaldo E.G. (2020). Association between oxidative stress and temporomandibular joint dysfunction: A narrative review. J. Oral Rehabil..

[12] Masahiro I., Shinya A., Nagata S., Miyata M., Hiroshi K. (2001). Relationships between perceived workload, stress and oxidative DNA damage. Int. Arch. Occup. Environ. Health.

[13] Soliman M.M., Aldhahrani A., Althobaiti F., Ahmed M.M., Sayed S., Alotaibi S., Shukry M., El-Shehawi A.M. (2022). Characterization of the Impacts of Living at High Altitude in Taif: Oxidative Stress Biomarker Alterations and Immunohistochemical Changes. Curr. Issues Mol. Biol..

[14] Crawford A., Fassett R.G., Geraghty D.P., Kunde D.A., Ball M.J., Robertson I.K., Coombes J.S. (2012). Relationships between single nucleotide polymorphisms of antioxidant enzymes and disease. Gene.

[15] Assavarittirong C., Samborski W., Grygiel-Górniak B. (2022). Oxidative Stress in Fibromyalgia: From Pathology to Treatment. Oxid. Med. Cell. Longev..

[16] Geyik S., Altunısık E., Neyal A.M., Taysi S. (2016). Oxidative stress and DNA damage in patients with migraine. J. Headache Pain.

[17] de Almeida C., Amenábar J.M. (2016). Changes in the salivary oxidative status in individuals with temporomandibular disorders and pain. J. Oral Biol. Craniofacial Res..

[18] Rodríguez de Sotillo D., Velly A.M., Hadley M., Fricton J.R. (2011). Evidence of oxidative stress in temporomandibular disorders: A pilot study. J. Oral Rehabil..

[19] Cai H.X., Luo J.M., Long X., Li X.D., Cheng Y. (2006). Free-radical oxidation and superoxide dismutase activity in synovial fluid of patients with temporomandibular disorders. J. Orofac. Pain.

[20] Vrbanović E., Alajbeg I.Z., Vuletić L., Lapić I., Rogić D., Andabak Rogulj A., Illeš D., Knezović Zlatarić D., Badel T., Alajbeg I. (2018). Salivary Oxidant/Antioxidant Status in Chronic Temporomandibular Disorders Is Dependent on Source and Intensity of Pain -A Pilot Study. Front. Physiol..

[21] Bouayed J., Rammal H., Soulimani R. (2009). Oxidative stress and anxiety: Relationship and cellular pathways. Oxid. Med. Cell. Longev..

[22] Rus A., Robles-Fernandez I., Martinez-Gonzalez L.J., Carmona R., Alvarez-Cubero M.J. (2021). Influence of Oxidative Stress-Related Genes on Susceptibility to Fibromyalgia. Nurs. Res..

[23] Pourvali K., Abbasi M., Mottaghi A. (2016). Role of Superoxide Dismutase 2 Gene Ala16Val Polymorphism and Total Antioxidant Capacity in Diabetes and its Complications. Avicenna J. Med. Biotechnol..

[24] Djokic M., Radic T., Santric V., Dragicevic D., Suvakov S., Mihailovic S., Stankovic V., Cekerevac M., Simic T., Nikitovic M. (2022). The Association of Polymorphisms in Genes Encoding Antioxidant Enzymes GPX1 (rs1050450), SOD2 (rs4880) and Transcriptional Factor Nrf2 (rs6721961) with the Risk and Development of Prostate Cancer. Medicina.

[25] Little J., Higgins J.P., Ioannidis J.P., Moher D., Gagnon F., von Elm E., Khoury M.J., Cohen B., Davey-Smith G., Grimshaw J. (2009). STrengthening the REporting of Genetic Association Studies (STREGA)--An extension of the STROBE statement. Genet. Epidemiol..

[26] Vandenbroucke J.P., von Elm E., Altman D.G., Gøtzsche P.C., Mulrow C.D., Pocock S.J., Poole C., Schlesselman J.J., Egger M. (2014). STROBE Initiative. Strengthening the Reporting of Observational Studies in Epidemiology (STROBE): Explanation and elaboration. Int. J. Surg..

[27] Ohrbach R., Spalj S., Katic V., Alajbeg I., Celebic A. Diagnostic Criteria for Temporomandibular Disorders: Assessment Instruments. Version 15 May 2016. [Dijagnostički Kriteriji za Temporomandibularne Poremećaje (DK/TMP) Instrumenti Procjene: Croatian Version 23 March 2021].

[28] Beard C., Hsu K.J., Rifkin L.S., Busch A.B., Björgvinsson T. (2016). Validation of the PHQ-9 in a psychiatric sample. J. Affect. Disord..

[29] Mughal A.Y., Devadas J., Ardman E., Levis B., Go V.F., Gaynes B.N. (2020). A systematic review of validated screening tools for anxiety disorders and PTSD in low to middle income countries. BMC Psychiatry.

[30] Barsky A.J., Goodson J.D., Lane R.S., Cleary P.D. (1988). The amplification of somatic symptoms. Psychosom Med..

[31] Bernstein R.E., Delker B.C., Knight J.A., Freyd J.J. (2015). Hypervigilance in college students: Associations with betrayal and dissociation and psychometric properties in a Brief Hypervigilance Scale. Psychol. Trauma.

[32] Vrbanović E., Zlendić M., Alajbeg I.Z. (2022). Association of oral behaviours frequency with psychological profile, somatosensory amplification, presence of pain and self-reported pain intensity. Acta Odontol. Scand..

[33] Zlendić M., Vrbanović E., Tomljanović M., Gall Trošelj K., Đerfi K.V., Alajbeg I.Z. (2023). Association of oral behaviours and psychological factors with selected genotypes in pain-related TMD. Oral Dis..

[34] Hu J., Zhou G.W., Wang N., Wang Y.J. (2010). GPX1 Pro198Leu polymorphism and breast cancer risk: A meta-analysis. Breast Cancer Res. Treat..

[35] Palmirotta R., Barbanti P., De Marchis M.L., Egeo G., Aurilia C., Fofi L., Ialongo C., Valente M.G., Ferroni P., Della-Morte D. (2015). Is SOD2 Ala16Val polymorphism associated with migraine with aura phenotype?. Antioxid. Redox Signal..

[36] Hong E.P., Park J.W. (2012). Sample size and statistical power calculation in genetic association studies. Genom. Inform..

[37] Levartovsky S., Msarwa S., Reiter S., Eli I., Winocur E., Sarig R. (2021). The Association between Emotional Stress, Sleep, and Awake Bruxism among Dental Students: A Sex Comparison. J Clin Med..

[38] Wieckiewicz M., Grychowska N., Wojciechowski K., Pelc A., Augustyniak M., Sleboda A., Zietek M. (2014). Prevalence and correlation between TMD based on RDC/TMD diagnoses, oral parafunctions and psychoemotional stress in Polish university students. Biomed Res. Int..

[39] Mirhashemi A., Khami M.R., Kharazifard M., Bahrami R. (2022). The Evaluation of the Relationship Between Oral Habits Prevalence and COVID-19 Pandemic in Adults and Adolescents: A Systematic Review. Front. Public Health.

[40] Winocur-Arias O., Winocur E., Shalev-Antsel T., Reiter S., Levartovsky S., Emodi-Perlman A., Friedman-Rubin P. (2022). Painful Temporomandibular Disorders, Bruxism and Oral Parafunctions before and during the COVID-19 Pandemic Era: A Sex Comparison among Dental Patients. J. Clin. Med..

[41] Alajbeg I.Z., Vrbanović E., Lapić I., Alajbeg I., Vuletić L. (2020). Effect of occlusal splint on oxidative stress markers and psychological aspects of chronic temporomandibular pain: A randomized controlled trial. Sci. Rep..

[42] Rajaraman P., Hutchinson A., Rothman N., Black P.M., Fine H.A., Loeffler J.S., Selker R.G., Shapiro W.R., Linet M.S., Inskip P.D. (2008). Oxidative response gene polymorphisms and risk of adult brain tumors. Neuro Oncol..

[43] Galasso M., Dalla Pozza E., Chignola R., Gambino S., Cavallini C., Quaglia F.M., Lovato O., Dando I., Malpeli G., Krampera M. (2022). The rs1001179 SNP and CpG methylation regulate catalase expression in chronic lymphocytic leukemia. Cell. Mol. Life Sci..

[44] Kido T., Sikora-Wohlfeld W., Kawashima M., Kikuchi S., Kamatani N., Patwardhan A., Chen R., Sirota M., Kodama K., Hadley D. (2018). Are minor alleles more likely to be risk alleles?. BMC Med. Genom..

[45] Papasavva M., Vikelis M., Siokas V., Katsarou M.S., Dermitzakis E.V., Raptis A., Kalliantasi A., Dardiotis E., Drakoulis N. (2023). Variability in oxidative stress-related genes (SOD2, CAT, GPX1, GSTP1, NOS3, NFE2L2, and UCP2) and susceptibility to migraine clinical phenotypes and features. Front. Neurol..

[46] Gentile G., Negro A., D’Alonzo L., Aimati L., Simmaco M., Martelletti P., Borro M. (2015). Lack of association between oxidative stress-related gene polymorphisms and chronic migraine in an Italian population. Expert Rev. Neurother..

[47] da Rocha T.J., Silva Alves M., Guisso C.C., de Andrade F.M., Camozzato A., de Oliveira A.A., Fiegenbaum M. (2018). Association of GPX1 and GPX4 polymorphisms with episodic memory and Alzheimer’s disease. Neurosci. Lett..

[48] Teimoori B., Moradi-Shahrebabak M., Razavi M., Rezaei M., Harati-Sadegh M., Salimi S. (2019). The effect of GPx-1 rs1050450 and MnSOD rs4880 polymorphisms on PE susceptibility: A case-control study. Mol. Biol. Rep..

[49] Ayuso P., García-Martín E., Agúndez J.A.G. (2021). Variability of the genes involved in the cellular redox status and their implication in drug hypersensitivity reactions. Antioxidants.

[50] Tang T.S., Prior S.L., Li K.W., Ireland H.A., Bain S.C., Hurel S.J., Cooper J.A., Humphries S.E., Stephens J.W. (2012). Association between the rs1050450 glutathione peroxidase-1 (C > T) gene variant and peripheral neuropathy in two independent samples of subjects with diabetes mellitus. Nutr. Metab. Cardiovasc. Dis..

[51] Charniot J.C., Sutton A., Bonnefont-Rousselot D., Cosson C., Khani-Bittar R., Giral P., Charnaux N., Albertini J.P. (2011). Manganese superoxide dismutase dimorphism relationship with severity and prognosis in cardiogenic shock due to dilated cardiomyopathy. Free Radic. Res..

[52] Khawaja S.N., Nickel J.C., Iwasaki L.R., Crow H.C., Gonzalez Y. (2015). Association between waking-state oral parafunctional behaviours and bio-psychosocial characteristics. J. Oral Rehabil..

